# The Errors of Radiography

**Published:** 1900-09-08

**Authors:** 


### THE ERRORS OF RADIOGRAPHY.

It is said to be bad manners to look a gift-horse in the mouth. Still, there are points one wants to know, and in regard to radiography—that great gift to surgery by which so much that was dark has been made clear and plain—it is especially necessary to inquire closely how far and in what circumstances it is to be trusted. The Röntgen rays had hardly been introduced to public notice when they were seized upon by medical men as the very thing wanted; the imagination of surgeons as well as of laymen was caught by the splendid opportunities which they offered, and, in the newness of what they displayed, no doubt unwarranted conclusions were drawn in many cases. One can hardly wonder that while surgeons were watching with interest, not unmingled with dismay, the revelations made in regard to fractures which they had thought well set, litigious patients and shyster lawyers quickly availed themselves of the new method, scenting "malpraxis" and "compensation" in the air. To scientific surgery radiography is all gain, but to the busy surgeon, with a varied clientèle, it has certainly been full of pitfalls. It becomes important, then, to inquire what sort of errors may occur in radiography which may influence the medico-legal aspect of a case. This has lately been done by Dr. Carl Beck,[1] in a paper read before the Society of Medical Jurisprudence of New York. He says it should never be forgotten that a so-called Röntgen-ray picture is by no means an ordinary photograph of an object, but a silhouette only —that is, a photograph of its shadow—and that to interpret such shadows properly a thorough knowledge of the normal anatomical relations of the tissues and of the sort of shadows they are capable of producing is required. The carpus, for example, is peculiarly apt to produce errors in the skiagraph, the tuberosities of the trapezium, scaphoid, &c., doubling the thickness of the carpus and causing dark shadows which might be mistaken for foreign bodies. Normal sesamoid bones also in the early days were sometimes misinterpreted. The greatest diagnostic difficulties are presented by joints, and the more complicated a joint is the more complicated will the skiagraphs of its various positions appear. In cases of fracture also there are often great difficulties, for in the entire absence of displacement it is sometimes only a very distinct skiagraph that will show the line of fracture clearly. In some cases of inter-articular fracture, again, great trouble may be met with in interpreting the appearances; and, further, it must be remembered that when abnormalities are present it is easy to mistake them for the results of accident or maltreatment, so that it is often necessary to compare the skiagram with one of the opposite side. Various cases are mentioned by Dr. Beck, showing the necessity of taking skiagraphs in more than one direction if anything like an accurate conception of the condition of affairs is to be obtained. While, however, radiography has its limitations, it is held that a distinct plate will always tell the truth, and that if accompanied by the registration of the details of the operation, viz., the source of the current, the length of spark, the intensity of the tube, the distance of the platinum disc from the plate, the position of the object, the kind of plate, and the time of exposure, it will be a valid document intelligible to the expert. Admitting a simple skiagram as evidence to be interpreted by a jury would, however, be quite another thing.

[1] New York Med. Rec., Aug. 25.

